# Non-bacterial Thrombotic Endocarditis Presenting as Cardiogenic Shock With Anterior ST Elevation Revealing an Occult Cholangiocarcinoma: A Case Report

**DOI:** 10.7759/cureus.117501

**Published:** 2026-10-05

**Authors:** Colin Hannesse, Francesca Chiara Della Casa, Selda Aydin, Pamela Baldin, Anja Kerschen

**Affiliations:** 1 Department of Cardiovascular Intensive Care, Cliniques Universitaires Saint-Luc, Brussels, BEL; 2 Department of Pathology, Cliniques Universitaires Saint-Luc, Brussels, BEL

**Keywords:** cholangiocarcinoma, ecmo, hiv, non-bacterial thrombotic endocarditis, st-elevation, trousseau syndrome

## Abstract

Non-bacterial thrombotic endocarditis (NBTE) associated with cholangiocarcinoma is rare, with only a few case reports described. The diagnosis is usually made after a thrombotic event or stroke. We report the case of a 44-year-old South African woman who presented with cardiogenic shock and ST elevation while receiving rivaroxaban for a recent pulmonary embolism. Autopsy revealed myocardial embolization secondary to NBTE associated with cholangiocarcinoma. This case highlights the value of point-of-care echocardiography in ruling out complications or alternative diagnoses in atypical presentations of acute coronary syndrome. Furthermore, it underscores that, in NBTE, anticoagulation with heparin is the preferred regimen.

## Introduction

Non-bacterial thrombotic endocarditis (NBTE) is a rare form of endocarditis characterized by sterile fibrin and platelet deposition on cardiac valves, forming vegetations without active infection [1]. It encompasses several clinicopathological entities, including marantic endocarditis, which is secondary to advanced malignancy and represents the leading cause of NBTE [2].

Clinically, NBTE often remains silent until systemic embolization occurs. Embolic events may involve the brain, spleen, kidneys, or peripheral arteries, whereas coronary embolism is less common. Because fever or elevated inflammatory markers can coexist, NBTE is frequently misdiagnosed as infective endocarditis, delaying appropriate management. In some cases, the definitive diagnosis is established only at autopsy [3]. Management consists of therapeutic anticoagulation with heparin and treatment of the underlying cancer [1].

## Case presentation

A 44-year-old South African woman was brought to the emergency department by the prehospital emergency response team with a presumed diagnosis of ST-elevation myocardial infarction (STEMI). She had called the emergency services 30 minutes earlier for typical chest pain.

Upon arrival, she appeared distressed and reported severe oppressive chest pain accompanied by excruciating left-leg pain. Her initial vital signs were pulse (56 beats/minute), blood pressure (77/31 mmHg), and oxygen saturation (97%) on room air. On physical examination, the patient was agitated but responsive, moving all four limbs spontaneously. Cardiac auscultation revealed a grade 2/6 systolic murmur maximal at the mitral area. The extremities were cold, with a thready left femoral pulse and a capillary refill time of four seconds. Pulmonary auscultation was clear bilaterally, and no carotid bruits were detected.

Her past medical history was notable for human immunodeficiency virus (HIV) infection with an undetectable viral load under emtricitabine/tenofovir/dolutegravir and arterial hypertension treated with bisoprolol and hydrochlorothiazide.

Two months earlier, she was started on rivaroxaban 20 mg daily for pulmonary embolism secondary to deep-vein thrombosis after a long-haul flight. The transthoracic echocardiogram performed at that time showed no significant abnormalities.

The electrocardiogram (ECG) in the emergency department demonstrated complete atrioventricular block with an infra-Hisian escape rhythm at 52 beats/minute. ST-segment elevation was present in leads I, aVL, and V2-V3, with ST-segment depression in V5-V6 (Figure 1).

**Figure 1:**
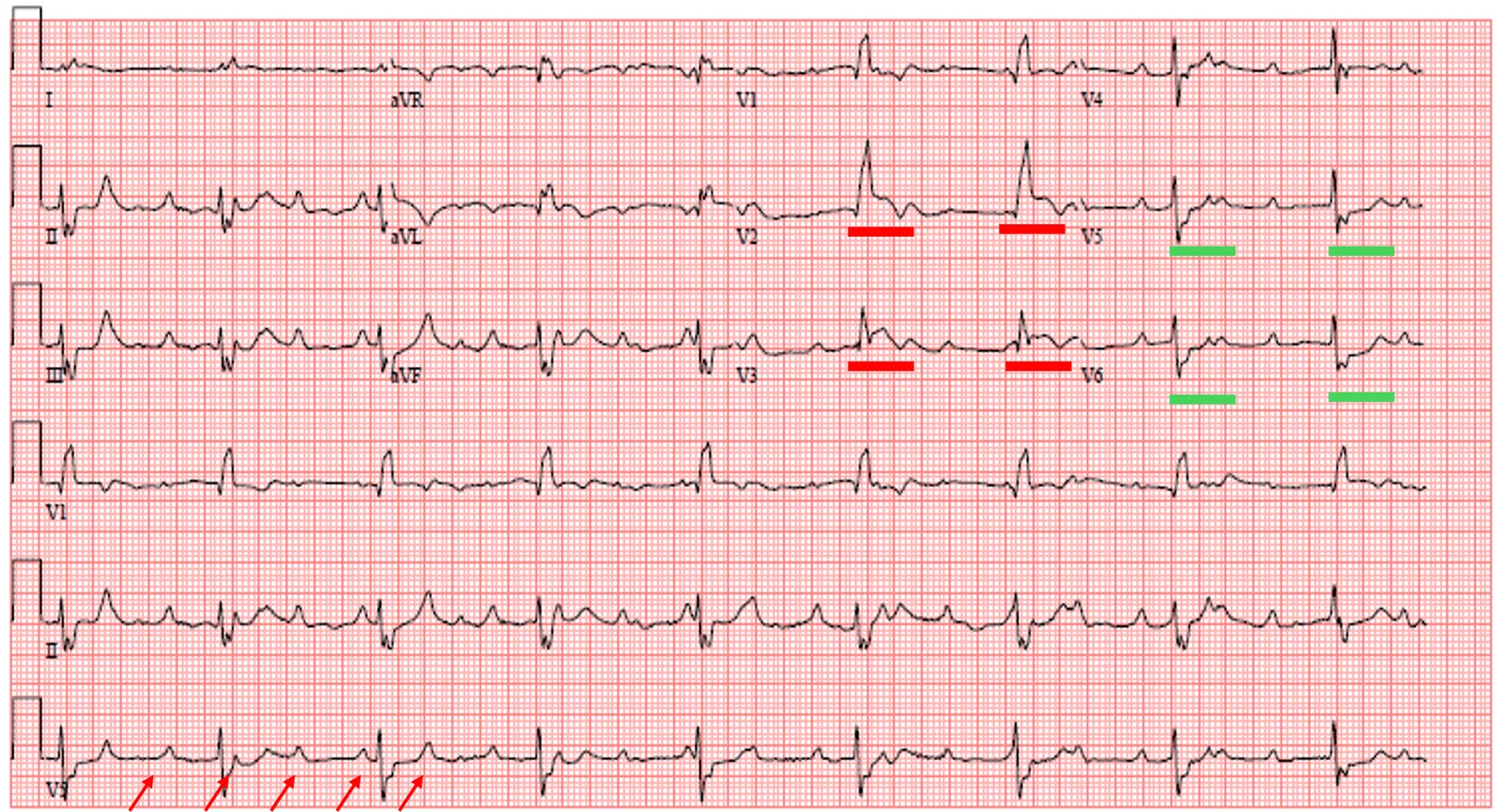
Electrocardiogram at initial presentation showing complete atrioventricular block with anterior ST-elevation myocardial infarction. Complete atrioventricular dissociation (red arrows) with ST elevation in V2-V3 (red lines) and ST-segment depression in V5-V6 (green lines). Paper speed: 25 mm/sec; Voltage: 10 mm/mVolt

Arterial blood gas showed a pH of 7.05, pCO_2_ of 16 mmHg, HCO_3_⁻ of 4 mmol/L, and lactate of 20 mmol/L. High-sensitivity troponin T was elevated at 132 ng/L (<10 ng/L), and creatine kinase was normal at 85 U/L (20-180 U/L), as were aspartate aminotransferase, alanine aminotransferase, and bilirubin.

Although the ECG was suggestive of anterior STEMI, the atypical clinical presentation prompted point-of-care transthoracic echocardiography to rule out aortic dissection. The study revealed extensive apical akinesia, marked thickening of both mitral and aortic valves, and a mobile echodensity attached to the atrial side of the anterior mitral leaflet, consistent with vegetation. There was also visually severe, central mitral regurgitation (Figures 2A-2C and Videos 1-2).

**Figure 2:**
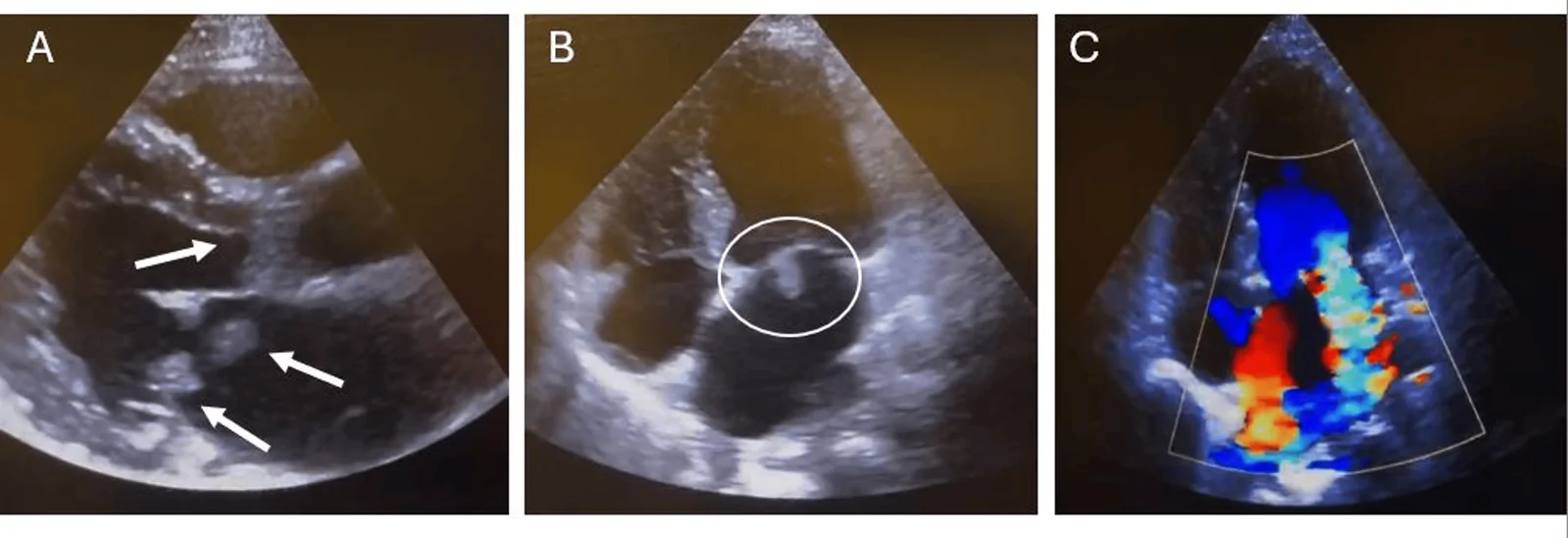
Point-of-care echocardiography. Aortic and mitral vegetations (A and B). Significant visual mitral regurgitation (C).

**Video 1:** Echocardiographic apical four-chamber view with and without color Doppler.

**Video 2:** Echocardiographic parasternal long-axis view with and without color Doppler.

Based on these findings, infective endocarditis complicated by coronary and left-lower-limb embolization was initially suspected.

Despite prompt medical management, the patient’s condition deteriorated rapidly. She developed severe bradycardia, and a non-invasive transcutaneous pacing attempt achieved electrical capture but failed to restore the arterial pulse. Cardiopulmonary resuscitation was initiated, and ventricular fibrillation was defibrillated to pulseless electrical activity. Given the patient’s young age, the in-hospital cardiac arrest, and her previously good functional status, the cardiogenic shock team decided to attempt extracorporeal cardiopulmonary resuscitation (ECPR). However, extracorporeal membrane oxygenation (ECMO) failed to generate an adequate flow, reaching a maximum of 2 liters/minute. Death was pronounced four hours after the onset of chest pain.

At autopsy, the heart weight was within the normal range. The coronary arteries appeared unremarkable upon dissection. A granular thrombus‑like material suggestive of vegetations was found on the leaflets of the mitral and aortic valves (Figures 3A, 3B).

**Figure 3:**
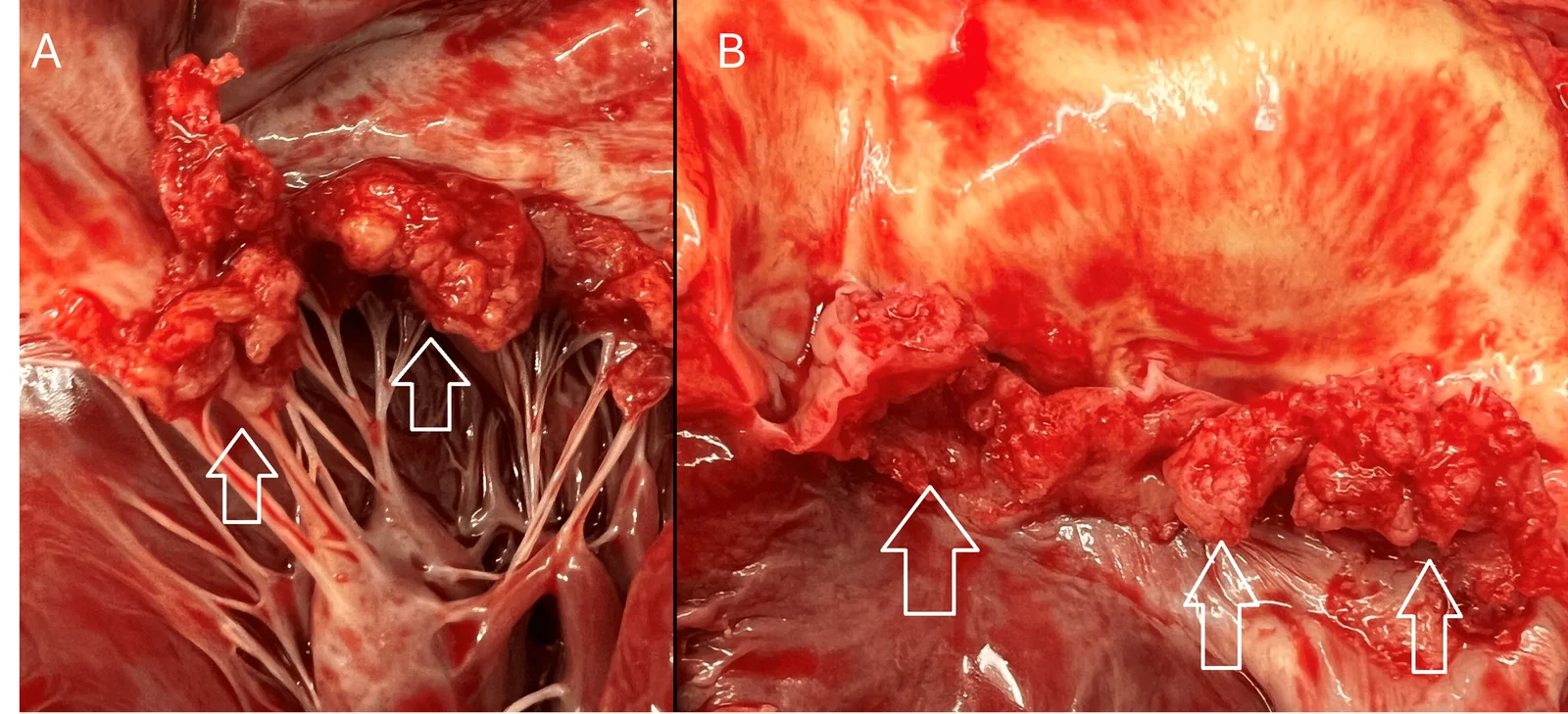
Autopsy findings: vegetations on the leaflets of the mitral (A) and aortic valves (B).

Upon histopathological examination, myocardial tissue samples showed no abnormalities, in particular no myocyte necrosis or hemorrhagic change. Numerous thrombi were found within the small‑caliber vessels of the myocardium (Figure 4).

**Figure 4:**
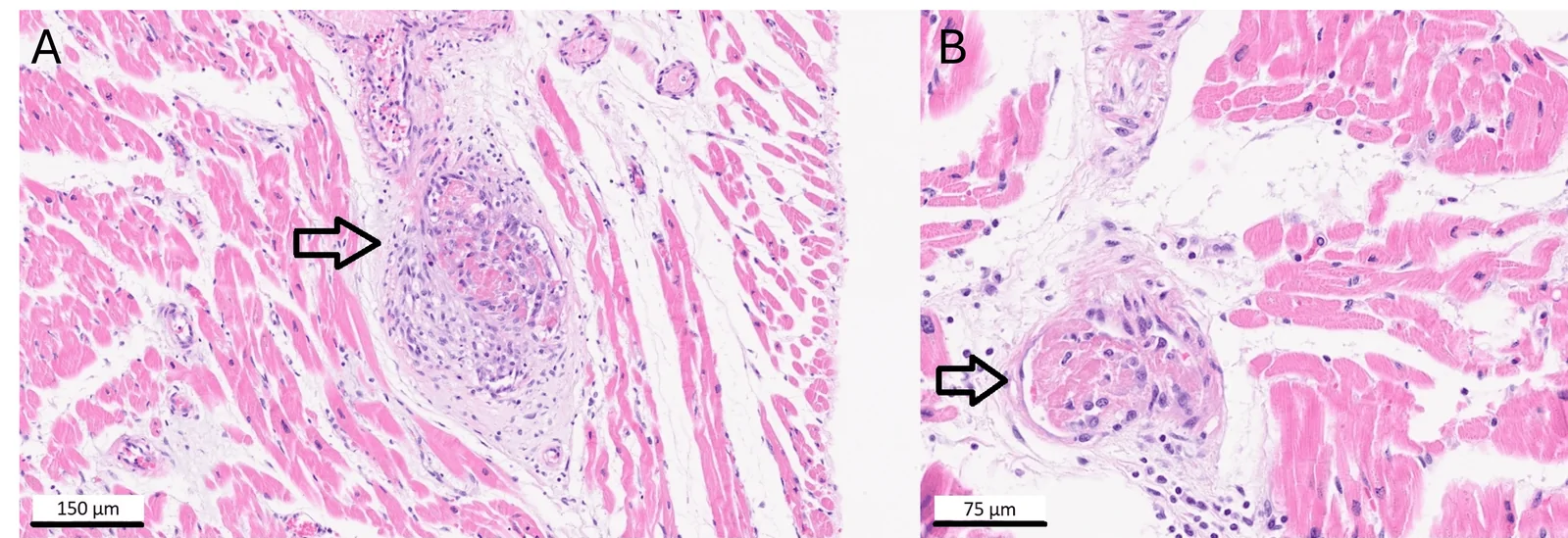
Histopathological examination of the heart: thrombi within small-caliber blood vessels of the heart (HES; 10× and 20× magnification, respectively). HES: hematoxylin and eosin staining.

Microscopically, the vegetations consisted of acellular material characterized by fibrinoid material associated with a few blood elements, adherent to the leaflets (Figure 5).

**Figure 5:**
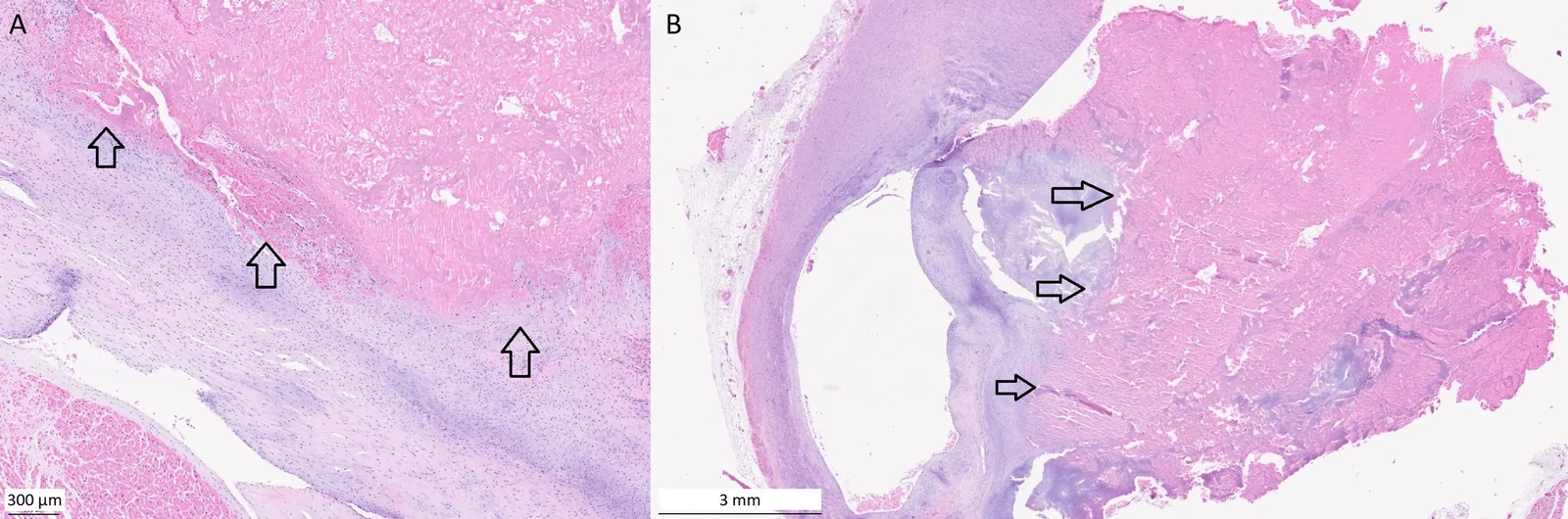
Histopathological examination of the vegetations. Acellular material consisting essentially of fibrinoid material associated with a few scattered blood elements. The arrows show the adherence of this acellular material to the mitral valve (A) and the aortic cuspids (B) (HES; 4.07× and 0.98× magnification, respectively). HES: hematoxylin and eosin staining.

On abdominal examination at autopsy, the liver contained multiple firm nodules, with the largest measuring 16 cm in diameter. Histopathologically, they consisted of glandular structures lined by cells with marked cytonuclear atypia. These cells extended along and infiltrated the bile duct, which showed pronounced epithelial irregularities with areas of both low-grade and high-grade dysplasia. Immunohistochemistry demonstrated cytoplasmic CK7 positivity in the tumor cells, supporting the diagnosis of cholangiocarcinoma (Figure 6).

**Figure 6:**
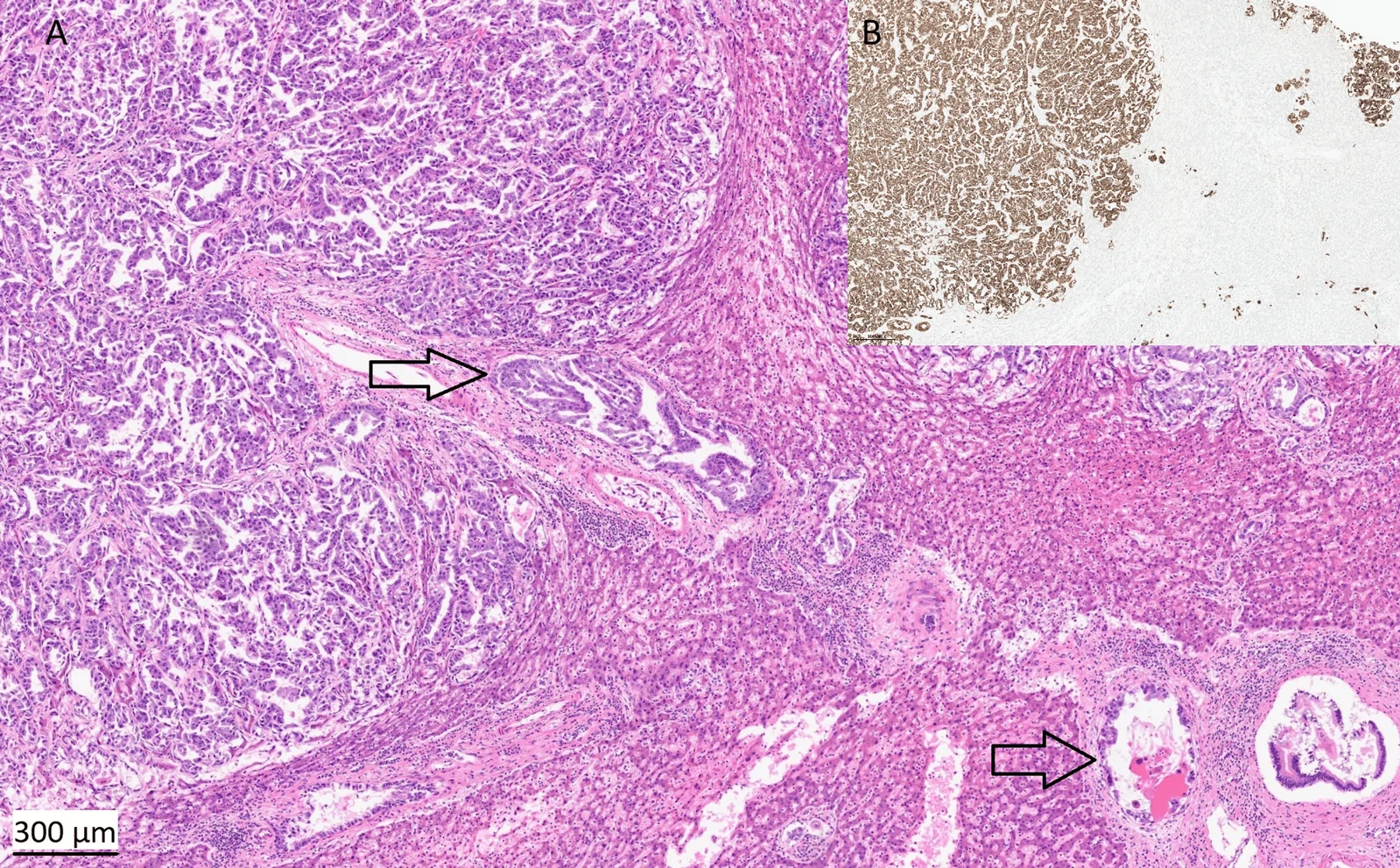
Histopathological examination of the liver. Neoplastic nodule characterized by glandular structures lined by highly atypical cells. Dysplasia of the lining epithelium in the portal tracts (A) (HES, 5× magnification). Immunohistochemistry for CK7 is positive (B) (Immunohistochemistry for CK7, 5× magnification). HES: hematoxylin and eosin staining.

Multiple whitish, firm nodules were also identified on the peritoneum. Histopathological examination revealed findings similar to those observed in the hepatic nodules, consistent with peritoneal carcinomatosis. The left femoral artery was not included in the examination. Therefore, an embolus at this site cannot be confirmed.

## Discussion

Cholangiocarcinoma-associated NBTE is rare [4], with only 10 cases reported in the literature and one with coronary embolism [5]. To our knowledge, this is the first reported case in which the initial presentation with cardiogenic shock led to the diagnosis of NBTE associated with cholangiocarcinoma.

For our patient, the combination of angina, elevated cardiac troponin, anterior ST-segment elevation, and new regional wall motion abnormality is consistent with the diagnosis of STEMI, suggesting complete occlusion of the left anterior descending coronary artery.

Nevertheless, autopsy revealed normal epicardial coronary arteries without obstruction, no myocardial necrosis, and multiple thrombi in the small-caliber vessels of the myocardium. The lack of myocardial necrosis and other structural changes is explained by the short interval, under four hours, between symptom onset and death [6].

We believe that the patient’s clinical presentation was caused by coronary embolization originating from the valvular vegetation. As NBTE vegetations are known to be friable [6], the absence of epicardial occlusion may be explained either by initial distal embolization in the microcirculation or by fragmentation of an epicardial embolus that occurred during cardiopulmonary resuscitation.

The causes of acute coronary syndrome secondary to endocarditis are largely related to coronary embolism, whereas coronary ostial obstruction is a far less common mechanism [7]. In our patient, coronary ostial obstruction would not explain the acute left-lower-limb pain, which we believe was caused by limb ischemia secondary to embolization of a vegetation. However, we cannot confirm this hypothesis because the femoral artery was not included in the forensic autopsy.

Alternative causes such as Takotsubo syndrome or severe systemic hypoperfusion are unlikely given the lack of specific electrocardiographic or echocardiographic findings, and they would not explain the atrioventricular block.

The value of point-of-care echocardiography in atypical presentations of acute coronary syndrome is illustrated by our case, as it can help rule out alternative diagnoses or acute complications, thereby guiding appropriate patient management and preventing inappropriate interventions.

As in this case, up to 10% of unprovoked venous thromboembolism diagnoses precede the detection of an underlying cancer [8]. These cancer-associated thrombotic events, called “Trousseau syndrome,” refer to the procoagulant state induced by tumors. Its clinical expression is a spectrum with NBTE in its most severe form [1]. The underlying mechanism involves endothelial injury and a hypercoagulable state that promotes sterile thrombus formation on valvular surfaces. Adenocarcinomas of the gastrointestinal tract and lung are the main cancers related to NBTE [3].

In suspected cancer-associated thrombosis, age-appropriate cancer screening, complemented by additional investigations based on the patient's history and physical examination in patients with unprovoked pulmonary embolism, is recommended [9]. This is mainly because extensive cancer screening has not been associated with a reduction in mortality [8, 9].

Given the aggressive nature of our patient's disease, it is unlikely that earlier screening would have had a substantial impact on her course. In any case, a long-haul flight combined with HIV was initially considered a reasonable cause of the pulmonary embolism.

HIV infection, even when adequately treated, is a well-established risk factor for thromboembolism with a two- to ten-fold increase of venous thromboembolism due to endothelial dysfunction, platelet activation, and coagulation activation [10]. This factor may have contributed to our patient's dramatic clinical presentation.

In NBTE, anticoagulation is advised to minimize the risk of embolic events [1]. Low-molecular-weight or unfractionated heparin is preferred over vitamin K antagonists or direct oral anticoagulants (DOACs), as evidence is limited and treatment failures have been reported [6]. This was the case in our patient, who was receiving rivaroxaban. Again, the procoagulant state induced by HIV may have contributed to the ineffectiveness of the DOAC.

## Conclusions

We report the first case of a coronary embolism secondary to NBTE associated with cholangiocarcinoma, diagnosed following a cardiogenic shock presentation. The diagnosis of NBTE can be challenging, as its clinical and echocardiographic features may overlap with those of infective endocarditis, potentially leading to misdiagnosis and delayed recognition. This diagnostic challenge is particularly relevant when NBTE presents as acute coronary syndrome. Coronary embolism secondary to endocarditis is an uncommon initial presentation and may not be immediately considered in the differential diagnosis. This case highlights the value of point-of-care echocardiography in patients presenting with atypical presentations of acute coronary syndrome to identify alternative diagnoses or complications to prevent inappropriate treatment. Furthermore, this case emphasizes that, in NBTE, the preferred regimen for anticoagulation is heparin, given the failure of anticoagulation despite ongoing DOAC therapy.
